# Supplementary material for: Risk factors for stillbirth and neonatal mortality among participants in Mobile WACh NEO pilot, a two-way SMS communication program in Kenya
Source: PLOS Glob Public Health. 2022 Jul 21;2(7):e0000812. doi: 10.1371/journal.pgph.0000812 (PMC10021995; doi:10.1371/journal.pgph.0000812)
Supplement: Attachment — Submitted filename: Response to Reviewers 15 APR 2022.docx [file pgph.0000812.s001.docx]

April 6, 2022

RE: Response to reviewers PLOS Global Health manuscript: **PGPH-D-21-00877 Risk Factors for Stillbirth and Neonatal Mortality Among Participants in Mobile WACh NEO, a Pilot Two-Way SMS Communication Program in Kenya**

Dear PLOS Global Health Editorial and Reviewer Teams :

Thank you for the very thorough and scientifically helpful review of our manuscript. We have addressed the comments one by one below and made corresponding changes to the manuscript with “track changes”. Both the “track changes” and “clean” version have been submitted.

**PGPH-D-21-00877
Risk Factors for Stillbirth and Neonatal Mortality Among Participants in Mobile WACh NEO, a Pilot Two-Way SMS Communication Program in Kenya
PLOS Global Public Health**

**Reviewer #1:** This manuscript would be a helpful contribution to the literature for understanding how mhealth could help to increase access to care for pregnant women and mothers in post-partum for improved and maternal outcomes. I hope the authors will find the comments below helpful as they revise the paper.

Background:
Lines 75-78, authors should be **clear about this study aim/research question**. Do you aim at assessing the effect of the “two-way SMS intervention” during pregnancy and post-partum on maternal and infant outcomes? Or your objective was just to describe these outcomes among the study participants?
Thank you for the opportunity to define the aims of this analysis and paper more clearly, which are separate from the aims of the parent demonstration project, Mobile WACh NEO pilot. The overall objective of the parent project was to determine the effect of this mHealth intervention on maternal and neonatal outcomes. The purpose of this paper and analysis is focused on determining maternal characteristics associated with neonatal death and stillbirth and describing participant messaging to study nurses prior to a stillbirth, infant hospitalization or infant death. In addition, we wanted to report on the type of SMS messaging that occurred prior to these events to inform future interventions.

- Lines 75-78 have been amended to: “We performed a secondary analysis on data from a pilot two-way SMS demonstration project (Mobile WACh NEO pilot) to determine maternal characteristics associated with neonatal death and stillbirth and describe participant messaging to study nurses prior to a stillbirth, infant hospitalization or infant death“
- The abstract has been updated to include that we “performed a secondary analysis of data from the Mobile WACH NEO longitudinal pilot study”.

Methods’ section:
2.) As you describe the intervention, include specific information about the expected speed of responding to the messages from mothers by study nurses. You should also summarize this information in the results’ section, as well as the number of messages sent during the non-working hours or the weekend.

- The methods have been updated to include *“The goal was to respond to every message within the same day if the message was sent during study clinic hours (8AM to 5pm, Monday to Friday). The message system did not capture response times to specific questions participants sent but standard procedures were to reply to urgent messages within an hour or by the end of the day if non-urgent. For those messages received off hours the procedure was to respond in the morning of the next day and Monday morning for weekend messages.”*
- The results have been updated to reflect: “*42.9% (8556/19,950) of all participant messages were sent off hours (outside 8AM to 5PM) or on the weekend.*”

3) You should include a **flowchart of the recruitment** of study participants, with a brief description of exclusion/inclusion criteria on each step.
a. Lines (134-135), you said that of the 3,108 women screened, 1,089 were eligible for this study, but only 798 were enrolled – what happened to the other 291 eligible women?

- Figure 1 (a CONSORT diagram) is now added. This figure reflects the reasons that women were not enrolled.

4) For the study inclusion criteria, you should explain the **rationale of choosing pregnant women at 28-36 weeks of gestation**. There is evidence that early initiation of antenatal care (<12 weeks gestation) is associated with improved pregnancy and birth outcomes, why not taking this window of opportunity?

We agree with the reviewers that earlier engagement with pregnant women would be optimal. Most women in this context, however, present in the late 2nd trimester for their first visit. The average gestational age at presentation in the clinic where we work is 23 weeks. Also, the logistics of the clinic flow, study and funding timeline as well as the period available for follow-up created limitations to when women could be recruited and followed. Therefore, this intervention was specifically designed to target the highest risk peripartum period for women and their newborns.

5) You will need to revise the statistical analysis part according to the study objectives. **If the study aim was to assess the effect of exposure** to the “two-way SMS intervention” on maternal and infant outcomes, then the expected analysis should be assessing whether there was an association between exposure to the intervention and outcomes, controlling for possible confounding factors, including socio-demographic characteristics.

The aims of this secondary analysis were to determine maternal characteristics associated with neonatal death and stillbirth and describe participant messaging to study nurses prior to a stillbirth, infant hospitalization or infant death. The objectives referred to by the reviewer were for the parent study (manuscript separate and in preparation) and not this analysis.

Results’ section:
6) In Tables 1&2, you should include at the top the total number of study participants (N) for each study site (rural/urban).

- This has been done.

  Discussion section:
  7) The **discussion section should be deeply revised** to reflect more of what should be expected from a classical discussion section:
  a) Include an introductory as the first paragraph for summarizing the study aim and all key findings.
  b) Interpret the key findings by comparing them with the previous literature
  c) Discuss the major limitations of this study, and how they should affect the interpretation of the findings. For example,
  d) Discuss the implications of your results as well/conclusions from your findings.

The discussion has been heavily revised to incorporate the helpful feedback above. Because it is so heavily revised, “track changes” was too messy to leave enabled.

8) There is no need to put much effort in comparing your study estimates to the other estimates reported by nationally representative surveys. Your study sample should never be expected to produce nationally representative estimates. However, you may want to discuss any comparisons under the study limitations.

Agreed and this has been done.

9) Lines 232-240, again, **why didn’t you adopt the WHO’s definition of stillbirth** when you defined the study inclusion criteria?

We agree with the reviewers that earlier enrollment and ability to capture the outcome of stillbirth by WHO criteria would be optimal. Most women in this population, however, present for antenatal care in the late 2nd trimester and therefore we were unable to capture all pregnancy outcomes starting at 28 weeks gestation.

10) Lines 263-265, I don’t understand the **following statement** and it is not suggested by your findings. “Our results suggest that prenatal identification of populations at high risk of perinatal death may not be an optimal method for targeting mHealth programs, and much like antenatal care programs they should instead be available to all pregnant women”.

This line has been removed and the conclusions refined.

a. Make sure that your **conclusions are supported by the study results**. So revised.

**Reviewer #2:** Thank you for inviting me to review this interesting research work in two public clinic in Nairobi, Kenya. This study aims to see the effect of introduction of mobile based communication to women during pregnancy and is definitely valuable in providing new evidence on effect of innovation on health care.
However, there are some major concerns in terms of intervention introduction process.

First, any innovation cannot be introduced in vacuum, it requires context, facilitator for successful implementation. Please see this important reference <https://implementationscience.biomedcentral.com/articles/10.1186/1748-5908-7-25>. The researcher have not provided **how the implementation was done**. Who were the facilitators to introduce the innovation, what was the process like.

The Mobile WACh NEO intervention, described in this analysis, was iteratively designed with formative work including a development workshop with members of the Kenya MOH Division of Reproductive Health, Kenyan medical professionals including those working in the clinics, programmatic partners working in MCH and digital health as well as technology partners. In addition, implementation improvements were made based on qualitative and quantitative evaluation of the first Mobile WACh project (Unger J, BJOG, 2018). This intervention was initiated in the clinics in partnership with country health officials and was introduced to the clinic staff with sensitization and flow mapping input to ensure successful implementation within the clinic services. The study procedures took place within the clinic but was staffed by designated research nurses.

We have now described within the manuscript the steps of study flow within clinics, lines 98-103.

Second, the flow figure does not cover the STROBE flow diagram. The 3,108 women screened and 1,089 eligible were not mentioned in the flow figure.

Figure 1 (a CONSORT diagram) is now added and includes the women screened and eligible.

Third, this study aims to provide the association of use of two way communication with the health outcomes. The researcher need to characterize the population who used the two way communication and those who didnot use two way communication using the Mobile WACh. The table 1 and 2 is redundant.

Thank you for the opportunity to clarify. This was a secondary analysis of a parent project. We did not aim to associate the messaging with outcomes, instead one of our aims was to associate maternal characteristics with outcomes and only describe, to inform future studies, the messaging that occurred around adverse events.

All participants received the SMS intervention, and 92% responded to messages- therefore we do not have a “did not use” group that can be reasonably compared with those that did message back to the study nurses.

Table 1 describes pre-enrollment characteristics for participants (demographics, phone access and pregnancy history) whereas table 2 describes characteristics and outcomes for participants during the study (messaging behavior, neonatal outcomes). We feel keeping these separate is most helpful for the results section.

Fourth, **the causal pathway i.e the DAG mapping needs to be explained in the method**. The expectation here is the two way communication effects on health outcome mental and mortality, I think there are a number of social, biological and health service factors which effect the mental health and mortality. This is not provided a causal pathway and **should be presented as multi-level analysis**.

We agree with the reviewer that there are many factors that influence perinatal outcomes. In this secondary analysis, however, we did not assess SMS use with health outcomes and therefore are not assessing causal pathways.

Fifth, I see most of the authors from US and only two from Kenya. Being a researcher and an advocate for decolonize global health, I think there should be a right balance of authorship. We need to promote researchers from global South.

We agree this is an important area to consider. The original author list had three authors from Kenya (Wandika, Wamalwa and Kinuthia) and we have added our data analyst Lusi Osborn consistent with the suggestions from the author reflexivity statement: https://associationofanaesthetists-publications.onlinelibrary.wiley.com/doi/10.1111/anae.15597.

Sixth, this study has merit, but needs a revision of tables as suggested above.

**Reviewer #3:** Thank you for submitting this well-written article. The study provides some interesting findings as it relates to the use and implications of mobile technology in improving birth outcomes, but the manuscript could benefit from an **expansion of the literature review**, as well as more details about the **implications of the study as it relates to policy**.

Agreed and this has been done.

- We cite additional references in the introduction re: existing evidence that messaging can be effective for peripartum support:
  - Lee SH. Effectiveness of mHealth interventions for maternal, newborn and child health in low- and middle-income countries: Systematic review and meta-analysis. J Glob Health. 2016
  - Feroz A. Role of mHealth applications for improving antenatal and postnatal care in low and middle income countries: a systematic review. BMC Health Serv Res. 2017

In the conclusions:

- (lines 304-309) “*effective interventions via two way SMS for this high-risk neonatal period may need to focus primarily on antenatal anticipatory guidance to engage women and families in early identification of neonatal illness when it later occurs and focus on connecting women to care quickly and at all times. Our ongoing randomized controlled study (Mobile WACh NEO RCT) incorporates these approaches and is in progress to determine the efficacy of this strategy to protect newborns and inform possible scale up of these programs*.”

**Journal Requirements:**

1. Please amend your detailed Financial Disclosure statement. This is published with the article, therefore should be completed in full sentences and contain the exact wording you wish to be published.

Please amend to “*Funding was provided by USAID Saving Lives at Birth, AID-OAA-F-16-00026, NIH/NICHD R01HD080460, NIH/NICHD 1R01HD098105 and NIH/NICHD K24HD054314. The funders had no role in study design, data collection and analysis, decision to publish, or preparation of the manuscript*.”

i). Please include all **sources of funding** (financial or material support) for your study. List the grants (with grant number) or organizations (with url) that supported your study, including funding received from your institution.

ii). State the initials, alongside each funding source, of each author to receive each grant.

*USAID (Saving Lives at Birth) AID-OAA-F-16-00026- KR, JK, JU, MB, DW, BW*

*NIH/NICHD R01HD080460- KR, JK, WJ, JU, MB, GJS, BW*

*NIH/NICHD 1R01HD098105- KR, JK, JU, DW, EC, AH, BW*

*NIH/NICHD K24HD054314- GJS*

iii). If any authors received a salary from any of your funders, please state which authors and which funders.

None

2. Please ensure that the **funders and grant numbers match** between the Financial Disclosure field and the Funding Information tab in your submission form. Note that the funders must be provided in the same order in both places as well.

Ok

3. Please **remove your Figure 1 legend** from the Supporting Information section of your manuscript.

This has been done
