# Supplementary material for: Risk factors for stillbirth and neonatal mortality among participants in Mobile WACh NEO pilot, a two-way SMS communication program in Kenya
Source: PLOS Glob Public Health. 2022 Jul 21;2(7):e0000812. doi: 10.1371/journal.pgph.0000812 (PMC10021995; doi:10.1371/journal.pgph.0000812)
Supplement: Attachment — Submitted filename: PLOS Global Public Health Reviewer Comments Response.docx [file pgph.0000812.s002.docx]

**June 16 2022**

RE: Response to reviewers PLOS Global Health manuscript: **PGPH-D-21-00877 Risk Factors for Stillbirth and Neonatal Mortality Among Participants in Mobile WACh NEO, a Pilot Two-Way SMS Communication Program in Kenya**

Dear PLOS Global Health Editorial and Reviewer Teams :

Thank you for the very thorough and scientifically helpful review of our manuscript. We have addressed the comments one by one below and made corresponding changes to the manuscript with “track changes”. Both the “track changes” and “clean” version have been submitted.

**Comments to the Author**

1. If the authors have adequately addressed your comments raised in a previous round of review and you feel that this manuscript is now acceptable for publication, you may indicate that here to bypass the “Comments to the Author” section, enter your conflict of interest statement in the “Confidential to Editor” section, and submit your "Accept" recommendation.

Reviewer #1: All comments have been addressed

Reviewer #2: All comments have been addressed

Reviewer #3: (No Response)

2. Does this manuscript meet PLOS Global Public Health’s [publication criteria](https://journals.plos.org/globalpublichealth/s/criteria-for-publication)? Is the manuscript technically sound, and do the data support the conclusions? The manuscript must describe methodologically and ethically rigorous research with conclusions that are appropriately drawn based on the data presented.

Reviewer #1: Yes

Reviewer #2: Yes

Reviewer #3: Yes

3. Has the statistical analysis been performed appropriately and rigorously?

Reviewer #1: Yes

Reviewer #2: Yes

Reviewer #3: Yes

4. Have the authors made all data underlying the findings in their manuscript fully available (please refer to the Data Availability Statement at the start of the manuscript PDF file)?

The [PLOS Data policy](https://journals.plos.org/globalpublichealth/s/data-availability) requires authors to make all data underlying the findings described in their manuscript fully available without restriction, with rare exception. The data should be provided as part of the manuscript or its supporting information, or deposited to a public repository. For example, in addition to summary statistics, the data points behind means, medians and variance measures should be available. If there are restrictions on publicly sharing data—e.g. participant privacy or use of data from a third party—those must be specified.

Reviewer #1: Yes

Reviewer #2: Yes

Reviewer #3: Yes

5. Is the manuscript presented in an intelligible fashion and written in standard English?

PLOS Global Public Health does not copyedit accepted manuscripts, so the language in submitted articles must be clear, correct, and unambiguous. Any typographical or grammatical errors should be corrected at revision, so please note any specific errors here.

Reviewer #1: Yes

Reviewer #2: Yes

Reviewer #3: No

**6. Review Comments to the Author**

**Reviewer #1:** Again, thank you for the opportunity to review the revised version of this paper. I would like to reiterate that this study will be a helpful contribution to the literature for understanding how mhealth could help to increase access to care for pregnant women and mothers in post-partum for improved and maternal outcomes. Authors have addressed most of my comments on the previous version, however below are a couple of minor comments on the current version:

1) For the study inclusion criteria, it would be important to explain the rationale of enrolling in this study pregnant women at 28-36 weeks of gestation. There is evidence that early initiation of antenatal care (<12 weeks gestation) is associated with improved pregnancy and birth outcomes, why not taking this window of opportunity? This comment on the previous version remains not addressed.

Thank you for this chance to clarify further in the manuscript. We have added more description about the intent of Mobile WACh NEO, an SMS intervention very specifically designed to address the issues and challenges faced in the period just before and after delivery. We designed this iteration of the project to target the highest risk period to women and infants to understand how this intervention might have an impact on decisions to deliver in a facility and identification and care seeking for early infant illness. Although we completely agree with early initiation of ANC and would like to design more studies to address this opportunity, this pilot was designed for only the small window around delivery.

The Methods now include:

*“This gestational age enrollment window was used because most obstetric patients present at these clinics late in the second trimester and the SMS intervention was specifically designed to address the highest risk period just before and after birth in order to potentially have the highest impact and be most feasible.”*

2) Lines 247-248. I think the following statement “Given the median enrollment age of 32 weeks, not all participants were monitored starting at the WHO defined stillbirth range of 28 weeks” doesn’t help to explain the discrepancy between the stillbirth/neonatal rates in this study and the national rates. The enrollment age shouldn’t affect the stillbirth rates, as the study only included pregnant women and followed up all of them to assess their outcomes and the study doesn’t report a significant loss-to-follow-up.

We agree being clear about how the study results compared to national data is important and we further expanded the paragraph in the discussion to specify which study outcomes varied from national:

*“Primary outcomes in our study of stillbirth (16 per 1,000 pregnancies) and perinatal death (36 per 1,000 pregnancies) were above Kenyan national rates of 13 and 29 respectively.[20] The rates may be higher than national rates due to closer monitoring for pregnancy outcomes in a research study. The study neonatal mortality (22 per 1,000 live births) was equal to Kenyan national rates.[20]”*

In respect to the question about under-estimation, we agree that rates should not change since women with stillbirths prior to 28 weeks would not be included in the total births. We have removed this phrase from the manuscript:

*­­­­­­­“…the study stillbirth rate is likely an under-estimation”*

**Reviewer #2:** Dear Authors

There have been much improvement in the revision. I still have some comments that the authors need to consider before final decision

1. This is a cohort study, so please use the standard epidemiological language rather than longitudinal study.

The text has been updated to reflect this important distinction (this analysis is a cohort study).

Abstract: *“We performed a secondary prospective cohort analysis of data from the Mobile WACh NEO ~~longitudinal~~ pilot study to determine maternal characteristics associated with neonatal death and stillbirth and examine participant messaging associated with these events.”*

Methods: *“This secondary analysis utilized data from the Mobile WACh NEO, a prospective cohort ~~demonstration project, a longitudinal~~ pilot study examining the effect of two-way SMS communication with healthcare workers (HCWs) on neonatal health outcomes.”*

2. One of the key finding is that the proportion women who had stillbirth messaged less frequently than women who had live birth. This relates well to the "Thaddeus and Mine" three delay model <https://doi.org/10.1016/S0277-9536(97)10018-1>. Women did not recognize the danger signs as a result there was delay in care. The NeoWatch technology helped to identify this important barrier to care. In that aspect, improving the use of the NeoWatch needs further contextualising the intervention. This needs to be reflected both in abstract and discussion.

We agree with the reviewer that the three delays model is important in considering the barriers women face to getting care when needed during pregnancy. However, in this study women who experienced stillbirth messaged more frequently in the antenatal period (adjusted RR for site 1.4, CI 1.0-1.96). The reality is that Mobile WACh NEO is addressing the first two delays but is limited to communication with women. As stillbirth is often a “silent” occurrence and while in some cases it is preventable, women may not be experiencing signs of symptoms they can identify. Therefore, the Mobile WACh intervention is quite limited in identifying conditions for which the woman or baby are not experiencing or noting a sign or symptoms which may limit its ability to impact most stillbirths.

The abstract has the additional line:

*“Messaging prior to stillbirth, while at similar levels as other periods, does not appear to have an impact as most women do not experience identifiable signs or symptoms prior to the event.”*

The discussion has this additional line:

*“Of note, almost a quarter of women who experienced a stillbirth did text during the week prior to the identification of the stillbirth. Stillbirth, while preventable in some cases, remains very difficult to predict, and often women do not experience identifiable signs and symptoms that could be addressed by the Mobile WACh counseling.”*

3. This still is a epidemiological study as you compared the risk factor for stillbirth and used messaging as exposure, so please use the STROBE checklist. CONSORT checklist is used in intervention study particularly if randomization to intervention is done.

We agree and have now verified items on the STROBE checklist for cohort studies v4 have been addressed.

The Results also refer to a “participant flow” diagram instead of a CONSORT diagram.

Reviewer #3: I have responded to the PLOS editorial questions above. have no additional comments

7. PLOS authors have the option to publish the peer review history of their article ([what does this mean?](https://journals.plos.org/globalpublichealth/s/editorial-and-peer-review-process#loc-peer-review-history)). If published, this will include your full peer review and any attached files.

**Do you want your identity to be public for this peer review?** If you choose “no”, your identity will remain anonymous but your review may still be made public.

For information about this choice, including consent withdrawal, please see our [Privacy Policy](https://www.plos.org/privacy-policy).

Reviewer #1: **Yes:** Alphonse Nshimyiryo

Reviewer #2: No

Reviewer #3: No
